# Case Report: Hermansky–Pudlak syndrome type 1 with pulmonary involvement in a 62-year-old Caucasian woman with rheumatoid arthritis

**DOI:** 10.3389/fimmu.2026.1778211

**Published:** 2026-04-27

**Authors:** Giacomo Giulianelli, Nicol Bernardinello, Elisabetta Cocconcelli, Stefania Rizzo, Cristina Basso, Antonella Bertomoro, Silvia Ferrari, Davide Colavito, Paolo Spagnolo, Elisabetta Balestro

**Affiliations:** 1 Respiratory Disease Unit, University Hospital, Padua, Italy; 2 Department of Cardiac, Thoracic, Vascular Sciences and Public Health; University of Padua, Padua, Italy; 3 Cardiovascular Pathology Unit, University Hospital, Padua, Italy; 4 Department of Medicine DIMED, Padova University Hospital, Padova, Italy; 5 R&I Genetics s.r.l., Padua, Italy

**Keywords:** albinism, case report, Hermansky-Pudlak syndrome, interstitial lung disease, rheumatoid arthritis, pulmonary fibrosis

## Abstract

**Introduction:**

Hermansky–Pudlak syndrome (HPS) is a rare autosomal recessive disorder characterized by oculocutaneous albinism (OCA), ocular nystagmus, bleeding diathesis and, in certain forms, pulmonary fibrosis (PF). We report a case of genetically confirmed HPS type 1 (HPS-1) with pulmonary involvement in a patient with rheumatoid arthritis (RA).

**Patient concerns and clinical findings:**

A 62-year-old Caucasian woman, former smoker, was referred for progressive pulmonary fibrosis (PPF) documented since 2019. History included chronic kidney disease and RA diagnosed in 2022, treated with hydroxychloroquine, prednisone, and abatacept. On presentation, she exhibited exertional dyspnea. Distinctive features included OCA, ocular nystagmus, and recurrent non-traumatic bruises, consistent with a bleeding tendency.

**Diagnostic assessment:**

Between 2021 and 2024, spirometry documented a decline in forced vital capacity - percent of predicted (FVC%) from 86% to 61% and a reduction in diffusing capacity of carbon monoxide - percent of predicted (DLCO%) from 60% to 37%. The six-minute walking test (6MWT) revealed exertional desaturation. High-resolution computed tomography (HRCT) showed PPF characterized by reticular thickening, ground-glass opacities (GGO), honeycombing, and traction bronchiectasis. Platelet aggregation study revealed impaired function with reduced CD41a expression, while transmission electron microscopy (TEM) confirmed rarefaction-to-absence of platelet dense granules. Genetic analysis identified a homozygous c.355del p.(His119ThrfsTer5) pathogenic variant in HPS1, confirming HPS-1.

**Interventions and outcomes:**

Abatacept was discontinued and rituximab initiated for RA management. Antifibrotic therapy was not pursued due to renal impairment and limited supporting evidence. Supportive measures included supplemental oxygen during exertion, pulmonary rehabilitation, and vaccination. Lung transplantation was deemed infeasible owing to renal failure and active autoimmune disease. The patient remains under follow-up.

**Conclusion:**

In patients presenting with PF, OCA, and bleeding diathesis, HPS should be suspected. Diagnostic workup begins with platelet aggregation study and TEM of platelets, and the diagnosis is confirmed by molecular genetic testing. No disease-modifying therapies exist for HPS-associated PF (HPS-PF); pirfenidone has uncertain benefit, while lung transplantation remains the only option for end-stage disease. This case underscores the need for early suspicion of HPS to guide investigations, counseling, and referral to specialized care. Moreover, the unusual association with RA is emphasized, as it may have contributed to PF.

## 1 Introduction

Hermansky-Pudlak syndrome (HPS) is an autosomal recessive disorder with an estimated prevalence of 1 in 500,000 to 1,000,000 individuals worldwide. It is characterized by oculocutaneous albinism (OCA), ocular nystagmus, and bleeding diathesis due to platelet dysfunction, while certain subtypes, particularly HPS-1 and HPS-4, are also associated with pulmonary fibrosis (PF) (1, 2).

Because of its rarity and multisystem involvement, HPS often poses a considerable diagnostic challenge, particularly when complicated by overlapping conditions that may themselves cause interstitial lung disease (ILD). This case is unique because it reports genetically confirmed HPS-1 with pulmonary involvement in a 62-year-old woman affected by rheumatoid arthritis (RA) and provides a stepwise description of a complex diagnostic pathway integrating clinical, functional, radiologic, hematologic, and genetic findings. By detailing this process, the report contributes to the limited literature on HPS and underscores the importance of recognizing syndromic clues such as albinism and bleeding diathesis in patients with PF.

## 2 Case description

A 62-year-old Italian woman was referred to our center in July 2024 for a progressive pulmonary fibrosis managed at the local hospital since 2019. During our visit, the patient reported worsening dyspnea with mild exertion, and occasional non-productive cough. The patient previously smoked heavily, both traditional and electronic cigarettes (21 pack-years for conventional cigarette smoking). She denied any significant occupational exposure, a family history of respiratory diseases or known hereditary disorders within the family. Her medical past condition included a chronic kidney disease of unknown origin, mild liver steatosis, and RA diagnosed in 2022 in accordance with the 2010 classification criteria jointly developed by the American College of Rheumatology and the European League Against Rheumatism (3). The patient presented with involvement of four small joints, a high-positive rheumatoid factor (RF), elevated erythrocyte sedimentation rate (ESR), and symptom duration exceeding six weeks, yielding a total score of 8 (according to the scoring system, “definite RA” can be classified with a score of 6 or greater); alternative conditions that could better account for the synovitis were excluded. First-line therapy with methotrexate was not initiated because of a contraindication related to the patient’s severe kidney failure and the presence of concomitant mild liver steatosis. Given the level of disease activity at treatment initiation (DAS28 = 4.65), a therapeutic regimen based on abatacept in combination with prednisone and hydroxychloroquine was selected.

**Figure 1:**
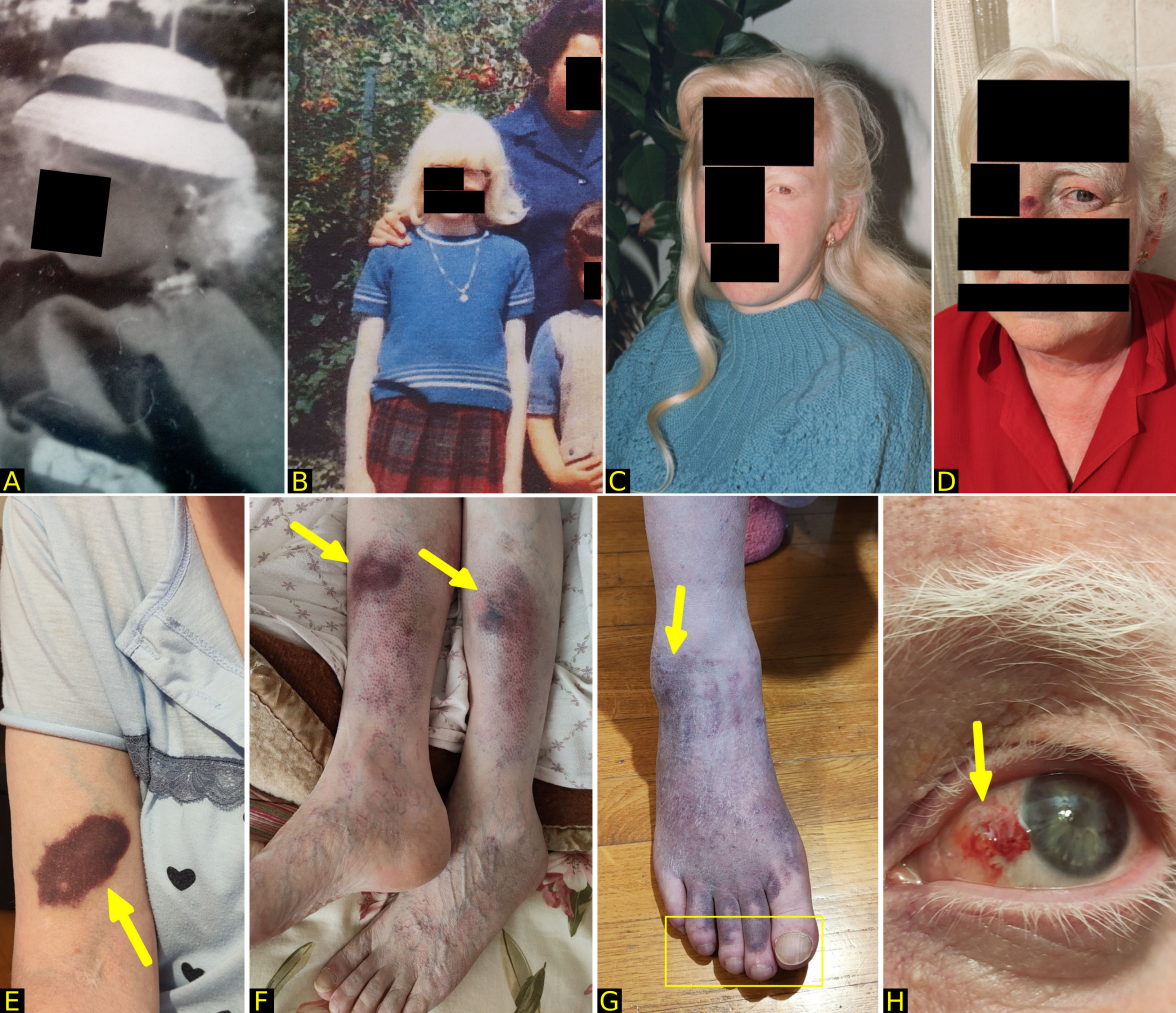
**(A-D)** oculocutaneous albinism, with evidence of hypopigmentation of hair, eyelashes, and eyebrows from childhood to the present day. In Figure **(B)**, the patient is shown with her mother (top right) and her sister (bottom right). **(E-H)** marked hemorrhagic diathesis with bruises affecting the arm after venipuncture **(E)**, lower limbs **(F)**, and feet **(G)** after a mild trauma, and eyes **(H)** after having lightly rubbed them due to itching (arrows). Note in Figure **(G)** the clubbing of the toenails (rectangle).

At the initial evaluation at our clinic in July 2024, the patient was eupneic at rest and had normal vital signs: temperature 36.6 °C, blood pressure 125/80 mmHg, heart rate 89 beats per minute, respiratory rate 20 breaths per minute, and oxygen saturation 96% on room air. On chest auscultation, vesicular breath sounds were heard on both sides, along with bibasilar crackles. Intriguingly, the patient presented OCA, which had been evident since birth, resulting in hypopigmentation of hair, skin, and eyes (confirmed by the ophthalmologist with iris transillumination) (**Figure 1**), associated with ocular nystagmus and multiple non-traumatic bruises on her arms. The patient reported a lifelong tendency to develop hematomas, occurring both spontaneously and after minor trauma (**Figure 1**).

## 3 Timeline

**Figure 2:**
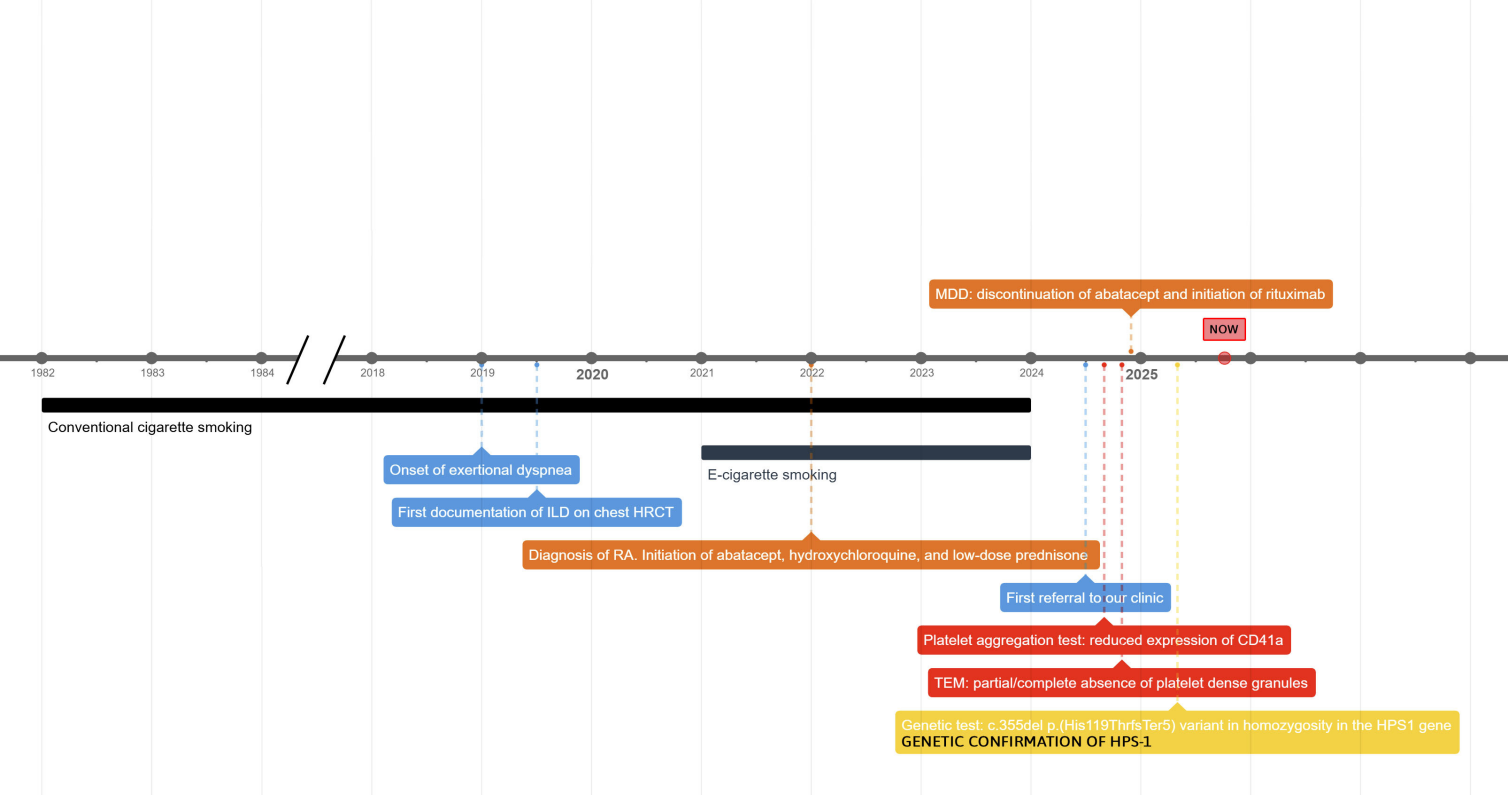
Timeline of primary diagnostic and therapeutic steps. HPS1/HPS-1, Hermansky-Pudlak syndrome type 1; HRCT, high-resolution computed tomography; ILD, interstitial lung disease; MDD, multidisciplinary discussion; RA, rheumatoid arthritis; TEM, transmission electron microscopy.

The timeline of primary diagnostic and therapeutic steps is provided in **Figure 2**.

## 4 Diagnostic assessment

**Figure 3:**
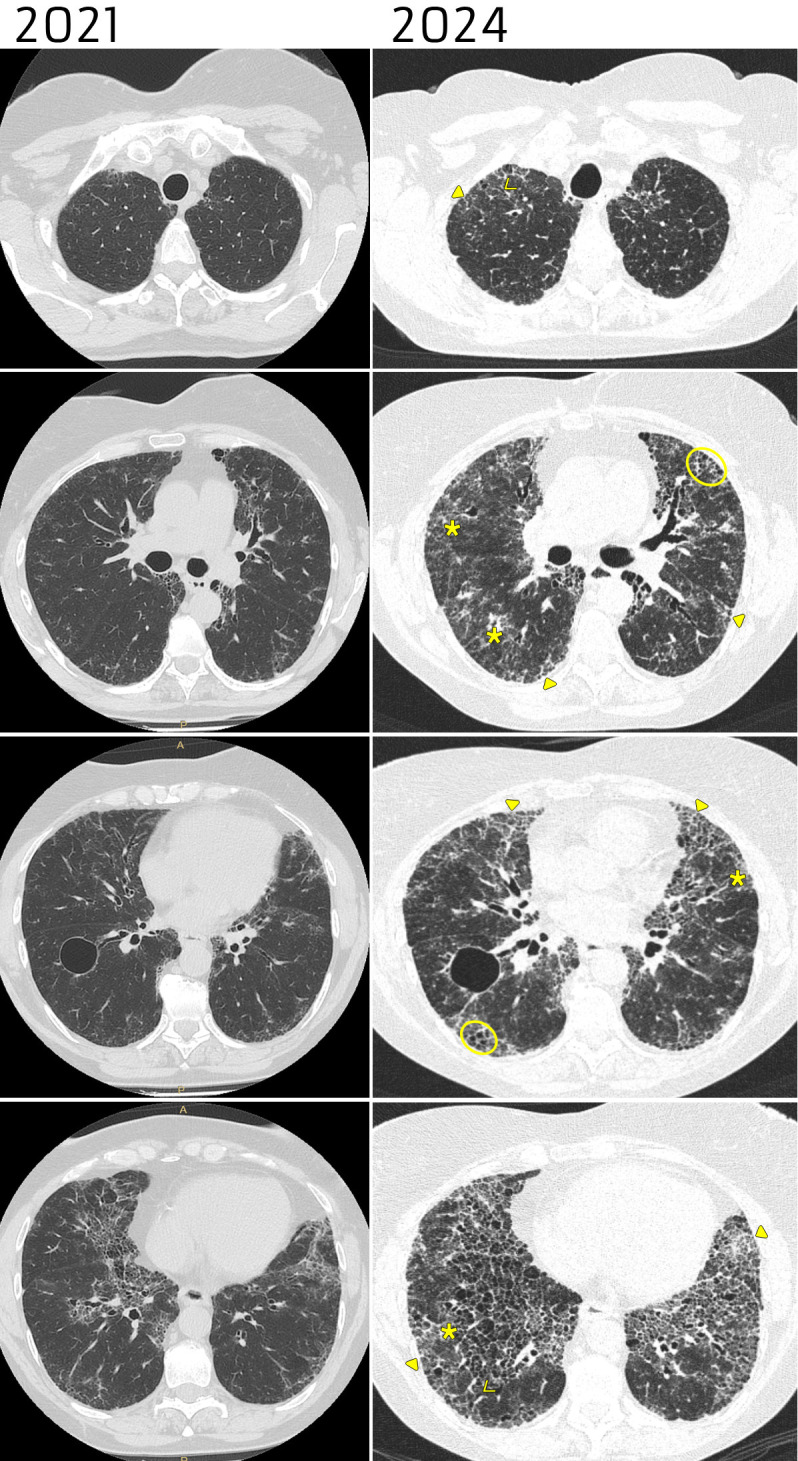
HRCT of the chest showing radiological progression from 2021 to 2024, with increased ground glass opacities and fibrosis. The 2024 scans show fibrosis characterized by reticular thickening of the interstitium (triangles), mild ground-glass opacities (asterisks), multiple traction bronchiectasis (arrows), and honeycombing (circles) with apicobasal gradient, configuring a UIP pattern.

Pulmonary function tests (PFTs) showed a decline in forced vital capacity (FVC) from 2.73 L (86%) in 2021 to 2.07 L (61%) in 2024 (-9% in forced vital capacity-percent of predicted - FVC% - in the last year), and in diffusing capacity of the lungs for carbon monoxide-percent of predicted (DLCO%), which decreased from 60% to 37% (-12% over the last year). The six-minute walking test (6MWT) revealed marked desaturation during exertion. High-resolution computed tomography (HRCT) showed reticular thickening of the lung interstitium, mild ground-glass opacities (GGO), multiple traction bronchiectasis, and honeycombing with apicobasal gradient, configuring a usual interstitial pneumonia (UIP) pattern. Thoracic imaging revealed a progressive increase in the extent of parenchymal alterations from 2019 to 2024. Disease progression between 2021 and 2024 is shown in **Figure 3**.

**Figure 4:**
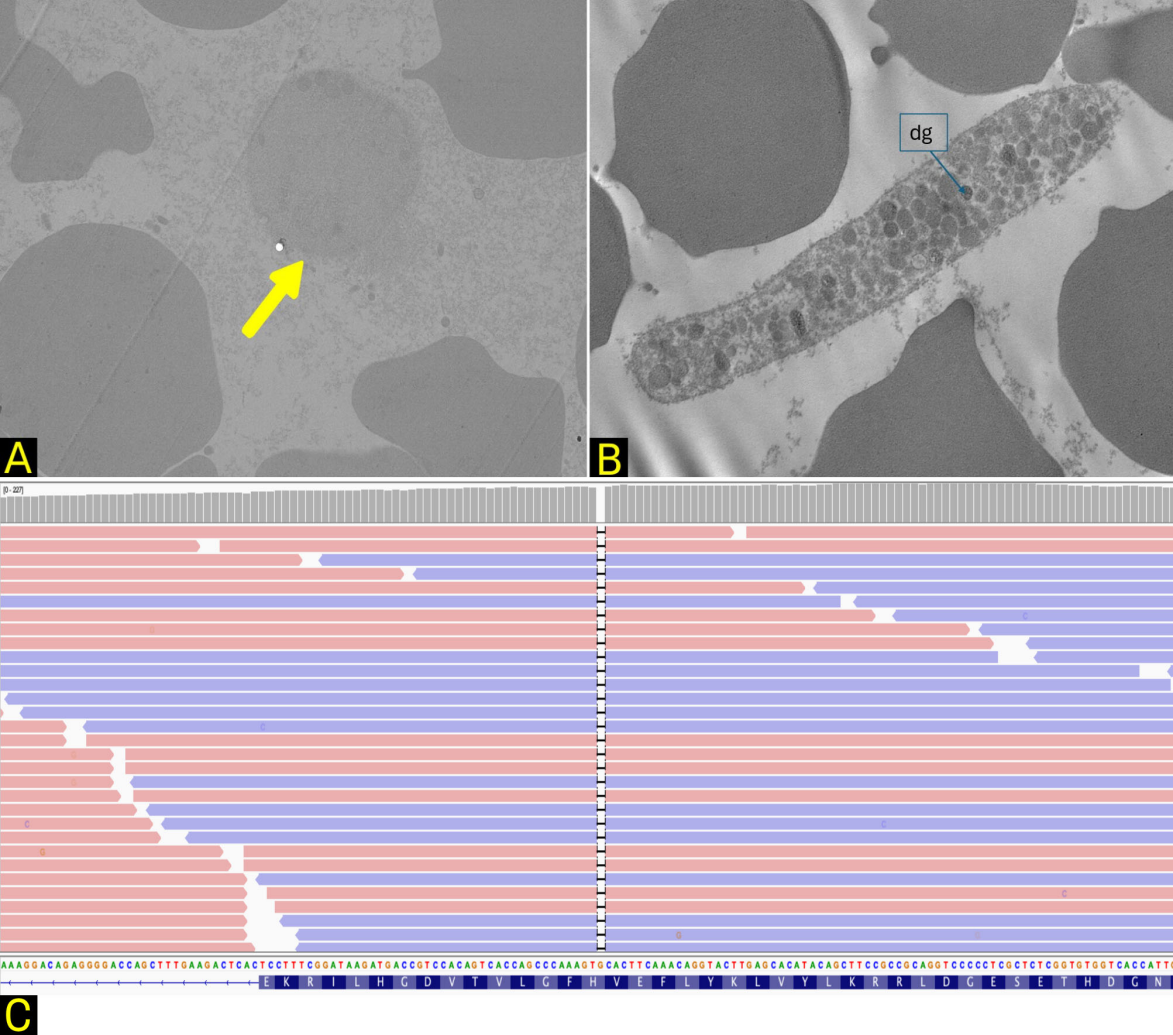
**(A, B)** whole blood analysis by TEM, showing platelets with rarefaction up to the disappearance of δ-granules (yellow arrow) in the patient **(A)**, as compared with a healthy control **(B)**. dg: δ-granules. **(C)** sequencing read showing the homozygous deletion.

The complete blood count revealed a platelet count within normal range. The patient underwent a platelet aggregation test, which showed an aggregation deficit and reduced expression of the platelet antigen GPIIb-IIIa (CD41a). Whole blood from the patient and a control underwent Karnovsky’s fixation (0.5% Karnovsky’s solution - 0.5% glutaraldehyde, 2% paraformaldehyde in 0.1M phosphate buffer, pH 7.3) and then Epon resin (Agar Scientific Ltd, Stansted, Essex, UK) embedding. For dehydration of the samples, a graded ethanol series and propylene oxide step was the method of choice for embedding in Epon. For polymerization we used a temperature of 45 °C for a period of 24–48 hours. After polymerization, trimming the block and cutting were performed with a diamond knife on the ultramicrotome (RMC Boeckeler, Tucson, AZ, USA) obtaining ultrathin (90 nm) sections, stained using uranyl acetate 5% and observed with a Hitachi H7800 (Hitachi, Tokyo, Japan) transmission electron microscope. Platelet analysis using transmission electron microscopy (TEM) revealed partial to complete absence of dense granules in the platelet cytoplasm (**Figure 4**). Finally, Whole Exome NGS sequencing (WES) analysis was performed from blood derived DNA extracted with Qiagen reagents according to manufactures instructions. WES library preparation was conducted with Agilent V8 Whole Exon Enrichment kit on Agilent Magnis dx automated pipettor. NGS sequencing was performed on an Illumina NextSeq2000 Sequencer (150 PE read sequencing). Reads alignment, as well as variant calling, was performed with GATK-based Dragen suite (Illumina). Variant prioritization and interpretation were performed with eVAI Software (enGenome). The genetic analysis highlighted the presence of the homozygous likely pathogenic c.355del p.(His119ThrfsTer5) variant in the Hermansky-Pudlak syndrome type 1 (HPS-1) gene (HPS1), known to be associated with autosomal recessive HPS-1 (**Figure 4**). The HPS1 variant c.355del p.(His119ThrfsTer5), also indexed as c.561del (rs281865075), has been reported in the literature in association with HPS (4).

The patient, who was seropositive for RA, with persistently elevated levels of RF and anti–cyclic citrullinated peptide (anti-CCP) antibodies (peak levels of 2098 kU/L and 172 kU/L, respectively), exhibited persistent moderate disease activity in the setting of progressive ILD. For this reason, the patient was evaluated for an adjustment of the rheumatologic treatment regimen. Following a multidisciplinary discussion, rituximab was selected as the therapeutic option due to the effect both on arthritis and on pulmonary involvement, under the assumption that RA may also have contributed to this condition. Low-dose prednisone and hydroxychloroquine were maintained. Prior to rituximab initiation, the risk of hypogammaglobulinemia was evaluated through quantitative serum protein analysis, which showed preserved immunoglobulin levels. Lymphocyte immunophenotyping showed increased CD8^+^ T cells; hematologic evaluation attributed this clonal T-cell lymphocytosis to the underlying rheumatologic disease, with no evidence of large granular lymphocytic leukemia and no contraindication to biologic therapy. Annual monitoring was recommended. Screening for viral hepatitis and tuberculosis (QuantiFERON-TB) was negative.

Nintedanib, a drug approved for the treatment of idiopathic pulmonary fibrosis (IPF) and progressive pulmonary fibrosis (PPF), and pirfenidone were avoided due to poor renal function and limited evidence supporting their use. At present, the patient is on supplemental oxygen during exercise. Influenza, SARS-CoV-2, and pneumococcal vaccinations and pulmonary rehabilitation program have also been recommended, along with reinforcement of the need to maintain abstinence from both conventional and electronic cigarette smoking.

Lung transplantation was considered not feasible due to concomitant kidney failure and RA requiring treatment.

From a pulmonological perspective, regular clinical and functional follow-ups have been scheduled. Using oxygen therapy, the patient attempts to manage dyspnea related to HPS-associated PF (HPS-PF) and to maintain an acceptable level of physical functioning, while taking measures to protect herself from respiratory infections. From a rheumatological standpoint, the patient has responded well to rituximab, with improvement in arthritis and a decrease in RA disease activity.

## 5 Discussion

This case illustrates the diagnostic and therapeutic challenges of HPS-1. The uniqueness of this case lies in its rarity and in the comprehensive documentation of a complex diagnostic pathway, from clinical suspicion to genetic confirmation.

HPS results from mutations affecting lysosome-related organelles (LROs). Defective melanosome function leads to OCA, while impaired platelet dense granule formation causes bleeding diathesis (5). In alveolar type II epithelial cells, dysfunction of Rab38—a small GTPase involved in surfactant homeostasis—alters lamellar body structure and disrupts surfactant regulation, thereby potentially contributing to the development of PF, although its precise role in the pathogenesis of fibrosis remains to be fully elucidated (1). Typically, middle-aged individuals with HPS-1 or HPS-4, as well as children and adults with HPS-2, are susceptible to also develop PF (6). According to a literature review by Vicary and colleagues, HPS-PF frequently manifests between 30 and 40 years of age (1). Inflammatory bowel disease-like manifestations occur in HPS-1 and HPS-4, while neutropenia is associated with HPS-2, HPS-9, and HPS-10 (7).

Laboratory tests generally show normal platelet counts and coagulation parameters but impaired aggregation on light transmission aggregometry. TEM, particularly the simplified “whole mount” technique, is considered the gold standard for evaluating the δ-granule content in platelets that are deficient in HPS (7). Definitive diagnosis of HPS is established through molecular genetic testing to identify pathogenic variants in one of the known HPS-associated genes. Genetic testing approaches include next-generation sequencing (NGS) (7). From the perspective of the pathogenic variant identified in the case described in our manuscript, this mutation has already been reported in the literature in patients with HPS. Sandrock et al. (4) described two Russian siblings, a male and a female aged 23 and 27 years, respectively, who presented with OCA, nystagmus, and a marked bleeding diathesis, including recurrent epistaxis and significant bleeding following tooth extractions; neither developed pulmonary fibrosis. In the male sibling, the diagnosis was established at the age of 4 years, considerably earlier than in the clinical case we report. A further point worth discussing is the potential impact of the different genetic backgrounds on HPS1 protein function and, consequently, disease severity. The Russian siblings described by Sandrock et al. were compound heterozygous for two truncating mutations, which may result in the production of differently truncated HPS1 proteins with variable residual effects on intracellular trafficking. In contrast, our patient carries homozygous frameshift mutation, which is expected to lead to severely truncated protein and likely complete loss of function. Previous studies suggest that the length and structural features of truncated HPS1 proteins may influence clinical phenotypes. In this context, the Russian siblings described by Sandrock et al. exhibited a more pronounced bleeding phenotype. This difference may reflect the distinct allelic combinations, as the presence of a longer truncated HPS1 protein in compound heterozygous patients could potentially interfere with intracellular trafficking to a greater extent than a uniformly truncated, likely non-functional protein. However, given the well-recognized clinical variability of HPS1, this genotype–phenotype correlation should be interpreted with caution. Finally, the relatively young age of the patients described by Sandrock et al. does not allow a comprehensive assessment of long-term disease severity, particularly regarding involvement of other organ systems. In this context, manifestations such as pulmonary fibrosis, which are known to occur later during HPS1, may not yet have developed and therefore require longitudinal follow-up.

Radiologically, HPS-PF is characterized on HRCT by GGO, reticulation, and in more advanced stages, traction bronchiectasis, lung volume loss, and honeycombing. Pleural thickening may also be present (8). Bronchoscopy is primarily used for research purposes and has not been demonstrated to have diagnostic value for HPS-PF (1). However, HPS-PF exhibits a histological pattern indistinguishable from UIP, which, when idiopathic, defines IPF (1, 9).

No effective treatments exist for HPS-PF, with over 70% of patients dying from disease complications (1). Corticosteroids provide no benefit and are not recommended (1). Patients should avoid cigarette smoke and irritants, prevent infections with adequate vaccination, and stay active to prevent deconditioning (1). Clinical trials evaluating the safety and efficacy of pirfenidone, a drug approved for the treatment of IPF, have shown mixed results. A randomized, placebo-controlled trial showed that pirfenidone is associated with reduced lung function decline in patients with mild or moderate HPS-PF (10). Moreover, three subjects who received open label pirfenidone for several years experienced mild functional and radiologic improvements with few side effects (11). Conversely, a second trial was discontinued due to futility (12). Lung transplantation remains the only therapeutic option for end-stage HPS-PF (1).

Literature identifies the UIP pattern as one of the most frequent radiologic patterns in rheumatoid arthritis-associated interstitial lung disease (RA-ILD). In a cohort study of 230 patients with RA-ILD, UIP was the predominant radiologic pattern (65%), followed by nonspecific interstitial pneumonia (NSIP) (24%). The presence of a UIP pattern and extensive lung involvement were associated with increased mortality (13). Consistently, histopathological studies have shown that the UIP pattern appears to be more prevalent than NSIP in this setting, and that patients with a UIP pattern frequently exhibited typical UIP features on HRCT (14).

Regarding RA control in this patient, the multidisciplinary meeting considered the introduction of rituximab appropriate due to its favorable pulmonary safety and efficacy profile. Considering that the rheumatologic disease at least contributed to the pulmonary involvement, this therapeutic choice is consistent with the 2023 American College of Rheumatology/American College of Chest Physicians guidelines for the treatment of ILD associated with systemic autoimmune rheumatic diseases (15). According to evidence from clinical trials and observational studies, rituximab represents a treatment of choice in RA-ILD, having been shown to improve or stabilize FVC, whereas other treatments like tocilizumab are considered second-line options.

A strength of this report is the detailed clinical, functional, radiologic, hematologic, and genetic characterization, which offers a comprehensive picture of HPS-1 with pulmonary involvement. Conversely, the concomitant presence of RA precludes a clear determination as to whether PF is entirely imputable to the underlying genetic alteration, whether the autoimmune disorder may have contributed, or whether both conditions acted synergistically, considering that the patient’s significant history of cigarette smoking may also have served as a contributing factor. This complexity may be regarded as another strength of our case report, as it could encourage further investigations, given that the coexistence of HPS and RA remains uncommon and poorly characterized in literature. Notably, the diagnosis of HPS was established late in life in this patient, underscoring the importance of maintaining a high index of suspicion and of accurately identifying specific subtypes of HPS, which may have important implications for anticipating and monitoring the development of organ involvement, as well as for prognosis.

Take-away lessons:

1. In a patient presenting with PF, OCA, and bleeding diathesis, HPS should be suspected.
2. Laboratory tests typically reveal a normal platelet count and standard coagulation parameters. The initial diagnostic step consists of a platelet aggregation study, which typically yields abnormal results.
3. TEM, especially the streamlined “whole mount” method, is regarded as the reference standard for assessing δ-granule content in platelets, which is lacking in HPS types 1 and 4.
4. A conclusive diagnosis of HPS is achieved via molecular genetic analysis, which detects pathogenic mutations in one of the HPS-related genes.
5. There are no therapies of proven efficacy for HPS-PF. Clinical research has explored the use of pirfenidone, though findings have been inconsistent. Lung transplantation is a potential therapeutic strategy for selected patients with end-stage lung fibrosis. Supportive therapy and the management of contributing comorbidities that may exacerbate pulmonary damage, such as RA in this case, are of utmost importance.

## 6 Patient perspective

From birth, I have lived with certain physical characteristics, such as fair skin and a tendency to develop bruise without an apparent cause, to which nystagmus was later added. When my breathing began to worsen, I initially attributed the fatigue to smoking; however, over time it became clear that something else was underlying my symptoms. This diagnostic journey ultimately led to a diagnosis of pulmonary fibrosis. Throughout the diagnostic process, I felt listened to and supported. The investigations were numerous and at times demanding, but I perceived that each step was aimed at achieving a deeper understanding of my clinical condition. The diagnosis of Hermansky–Pudlak syndrome was the formal recognition of a condition I had lived with for 60 years without knowing its name. Assigning a label to my daily experience did not represent a substantial change from my perspective, particularly considering the absence of definitive therapeutic options. The diagnosis of rheumatoid arthritis added a further layer of complexity, which was managed through close collaboration among the various specialists involved.

Awareness of pulmonary involvement and progressive respiratory limitation is difficult to accept, especially given the limited therapeutic options, further constrained by my renal insufficiency. I am currently being treated with low-dose corticosteroids, hydroxychloroquine, and rituximab, which have allowed satisfactory control of rheumatoid arthritis. Today, I live with greater awareness of my body and my condition. I manage dyspnea through oxygen therapy and aim to reduce the risk of infection by adopting appropriate preventive measures. I face daily life with the goal of preserving the best possible quality of life, within the limits imposed by the disease.

## 7 Informed consent

The patient subject of the present work provided her informed consent for the publication of her clinical history, data, and multimedia content related to herself.

## Data Availability

The raw data supporting the conclusions of this article will be made available by the authors, without undue reservation.
